# Belowground carbon allocation, root trait plasticity, and productivity during drought and warming in a pasture grass

**DOI:** 10.1093/jxb/erad021

**Published:** 2023-01-14

**Authors:** Manjunatha H Chandregowda, Mark G Tjoelker, Elise Pendall, Haiyang Zhang, Amber C Churchill, Sally A Power

**Affiliations:** Hawkesbury Institute for the Environment, Western Sydney University, Locked Bag 1797, Penrith, NSW, 2751, Australia; Hawkesbury Institute for the Environment, Western Sydney University, Locked Bag 1797, Penrith, NSW, 2751, Australia; Hawkesbury Institute for the Environment, Western Sydney University, Locked Bag 1797, Penrith, NSW, 2751, Australia; Hawkesbury Institute for the Environment, Western Sydney University, Locked Bag 1797, Penrith, NSW, 2751, Australia; Hawkesbury Institute for the Environment, Western Sydney University, Locked Bag 1797, Penrith, NSW, 2751, Australia; Department of Ecology, Evolution and Behaviour, University of Minnesota, 140 Gortner Laboratory, 1479 Gortner Ave, St. Paul, MN 55108, USA; Hawkesbury Institute for the Environment, Western Sydney University, Locked Bag 1797, Penrith, NSW, 2751, Australia; Brookhaven National Laboratory, USA

**Keywords:** *f*_BNPP_, non-structural carbohydrates, resilience, resistance, root and crown nitrogen, root crowns, root mass fraction, root tissue density, specific root length

## Abstract

Sustaining grassland production in a changing climate requires an understanding of plant adaptation strategies, including trait plasticity under warmer and drier conditions. However, our knowledge to date disproportionately relies on aboveground responses, despite the importance of belowground traits in maintaining aboveground growth, especially in grazed systems. We subjected a perennial pasture grass, *Festuca arundinacea*, to year-round warming (+3 °C) and cool-season drought (60% rainfall reduction) in a factorial field experiment to test the hypotheses that: (i) drought and warming increase carbon allocation belowground and shift root traits towards greater resource acquisition and (ii) increased belowground carbon reserves support post-drought aboveground recovery. Drought and warming reduced plant production and biomass allocation belowground. Drought increased specific root length and reduced root diameter in warmed plots but increased root starch concentrations under ambient temperature. Higher diameter and soluble sugar concentrations of roots and starch storage in crowns explained aboveground production under climate extremes. However, the lack of association between post-drought aboveground biomass and belowground carbon and nitrogen reserves contrasted with our predictions. These findings demonstrate that root trait plasticity and belowground carbon reserves play a key role in aboveground production during climate stress, helping predict pasture responses and inform management decisions under future climates.

## Teaser

Drought and warming can reduce forage production by altering belowground carbon allocation. However, a trade-off between belowground production and root trait plasticity can offset negative effects on aboveground plant productivity.

## Introduction

Global climate models predict an increase in mean surface temperature ranging from 2.6 °C to 4.8 °C by the end of this century, along with an increase in the intensity and frequency of drought conditions (Dai, 2011; Cowan *et al.*, 2014; IPCC, 2021). Empirical data suggest a reduction in grassland productivity, including pastures and rangelands, in response to increased variability in the climate, with the response varying from minimal, transient effects to long-lasting impacts (Dukes *et al.*, 2005; Wheeler and von Braun, 2013; FAO, 2018; Raza *et al.*, 2019). Such variability in ecosystem responses is ­probably linked to differences in a plant’s ability to withstand stress (resistance) and the time taken to recover from it (resilience) by adjusting trait values, termed trait plasticity (Volaire *et al.*, 2014).

Plant functional traits and their plasticity can illustrate multiple aspects of plant strategies, describe how individual species cope with heterogeneous environments, and provide a means of predicting patterns of plant responses to changes in their environment (Soudzilovskaia *et al.*, 2013; Funk *et al.*, 2017). For instance, under conditions such as inadequate nutrient supply and water stress, plants can alter their root traits, by increasing specific root length (SRL; root length per unit root dry mass) or reducing root tissue density (RTD; root mass per unit volume) or mean root diameter, to enhance soil resource uptake and maintain plant growth (Bruelheide *et al.*, 2018; Wieczynski *et al.*, 2019; Chandregowda *et al.*, 2022a). Plants can also alter biomass allocation to facilitate the acquisition of growth-limiting resources. Under soil resource limitation, plants favour biomass allocation to roots by increasing the fraction of belowground net primary production (*f*_BNPP_; belowground net primary production to total plant production ratio) (Hui and Jackson, 2006; Xu *et al.*, 2012; Chandregowda *et al.*, 2022a) and the root mass fraction (RMF; root biomass to total plant biomass ratio) (Poorter *et al.*, 2012), and shifting root production to deeper soil depths (Gibson-Forty *et al.*, 2016; Arndal *et al.*, 2018). Given the explanatory power of plant functional traits and the potential for trait plasticity (Grime and Mackey, 2002; Arnold *et al.*, 2019), there is considerable interest in using a traits-based approach to understand and predict plant species’ responses to climatic factors (Lozano *et al.*, 2020). Despite the importance of belowground (i.e. root) traits in reducing the impacts of extreme environments on plant growth, to date most of our knowledge of trait plasticity in response to climate extremes has relied disproportionately on aboveground traits (Bardgett *et al.*, 2014; de Vries *et al.*, 2016).

In addition to large inter- and intraspecific variability in individual root traits, there may be high coordination and trade-offs among morphological, physiological and chemical traits (Carmona *et al.*, 2021). For instance, thin fine roots with higher SRL are often correlated with lower root diameter, lower RTD, and higher tissue metabolic activity (Tjoelker *et al.*, 2005; Chevin and Hoffmann, 2017; Chandregowda *et al.*, 2022a). Thicker roots are typically associated with higher starch storage, which may support growth and maintenance as well as provide osmolytes important for hydraulic and tissue metabolic functions (Zwicke *et al.*, 2015). Phenotypic plasticity and covariation in the morphological, chemical and physiological traits of fine roots along the continuum of the root economic spectrum (Pérez-Ramos *et al.*, 2013, 2019; de la Riva *et al.*, 2021) may play a crucial role in resistance and resilience during drought and warming.

Resistance and resilience are two important aspects of plant responses to environmental stress, with the latter having received relatively little attention. Perennial grasses exhibit resource-conservative strategies under unfavourable growth conditions via the temporary senescence of aboveground shoots and translocation of non-structural carbohydrates (NSCs) and nitrogen (N) to root and crown storage. In grasses, the crown is the tissue that joins the roots with the shoots, bears shoot meristems and stores carbon (C) and N reserves (White, 1973; Piper and Paula, 2020). These belowground NSC and N reserves are critical in forming new tillers and building new photosynthetic tissues that drive recovery after climate stress or disturbance (e.g. grazing or fire). However, the degree to which plants accumulate and store NSCs and N is, in turn, moderated by environmental conditions via their effects on C assimilation and tissue maintenance (Sala *et al.*, 2012; Du *et al.*, 2020).

An increase in the intensity and frequency of drought in a future climate is predicted to reduce natural and managed grassland production in many ecoregions by negatively affecting plant physiological functioning (IPCC, 2013; Bodner and Robles, 2017). However, grasses can respond to these changes in climate by shifting their trait values along the continuum of the root economic spectrum to reflect either drought avoidance or tolerance strategies (da Silveira Pontes *et al.*, 2015; Basu *et al.*, 2016). Drought avoidance, the ability to maintain tissue water content despite declining water availability, can occur in grasses via increased investment in biomass allocation to roots and increasing acquisitive root trait values, which help to improve water acquisition. At the other end of the root economic spectrum, drought tolerance in grasses occurs via greater investment in resource-conservative traits, allowing plants to survive and recover from drought by maintaining hydraulic conductance and root metabolic activity in drying soil (Brunner *et al.*, 2015; Keep *et al.*, 2021).

Warming can limit plant performance either directly by determining the rate of photosynthesis and respiration (Lambers *et al.*, 2008) or indirectly through increasing water loss associated with temperature-induced increases in evapotranspiration (Park Williams *et al.*, 2013). In addition, a warmer environment can increase the atmospheric vapour pressure deficit, which may result in plant-regulated reductions in stomatal conductance to minimize water loss through transpiration (Cowan and Farquhar, 1977; Shaver *et al.*, 2000). While only a limited understanding of belowground responses to warming exists, root responses to experimental warming appear inconsistent. Reported effects of warming on root biomass and belowground net primary production (BNPP) include increases (Arndal *et al.*, 2018; Salazar *et al.*, 2020), decreases (Bai *et al.*, 2010; Arndal *et al.*, 2018), and no change (Carrillo *et al.*, 2014; Mueller *et al.*, 2018). As with plant growth and productivity, fine root traits show marked variation along temperature gradients (Freschet *et al.*, 2017; Huasco *et al.*, 2021). Moreover,  J. Wang *et al.* (2021) highlighted that the magnitude, duration, and method of warming could influence root responses to elevated temperature.

In addition to the independent effects of drought and warming on plant performance, global climate models also predict their co-occurrence in many ecoregions (Dai, 2011; Boeck *et al.*, 2016; IPCC, 2021). Warming during drought can exacerbate the effects of drought stress on soil water content by increasing evapotranspiration, and species’ responses to one stress may be modified by another, making it challenging to predict the direction and magnitude of responses to multiple stressors (Teskey *et al.*, 2015; Birami *et al.*, 2018). Water limitation can influence the temperature sensitivity of plant growth by reducing thermal optima. Kumarathunge *et al.* (2020) showed that warming negatively affects root growth more strongly relative to leaf or shoot growth during reduced water availability. On the other hand, J. Wang *et al.* (2021) distinguished the positive effect of warming on fine-root biomass in colder and drier climates in their global synthesis of fine-root responses to experimental warming. A recent meta-analysis of 1119 manipulative experiments reported that the warming effect on RMF switched from positive in dry areas to negative in wet areas, consistent with greater investment in belowground biomass under environmental conditions that are both hot and dry (Song *et al.*, 2019). However, these patterns are not universal (Kuster *et al.*, 2013), and may depend on prevailing climatic conditions or species identity (Zhou *et al.*, 2020).

A large proportion of temperate grasslands occur in areas that experience temperatures well above their thermal optima for productivity, such that rising temperatures may significantly reduce their growth (Cui *et al.*, 2006; Cullen *et al.*, 2009). Temperate grasses, in particular, may be especially vulnerable to higher temperatures owing to their lower thermal optima (Cui *et al.*, 2006; Munson and Long, 2017). In addition to warming, climate models predict that many ecoregions around the globe will be subject to reductions in cool-season (winter and spring) rainfall (FAO, 2015; CSIRO, 2020; Abram *et al.*, 2021). Here, we sought to address how the widely grown temperate perennial pasture grass *Festuca arundinacea* (Brown and Morgan, 1980; Kørup *et al.*, 2018) adjusts biomass allocation and root morphological and chemical traits in response to drought, warming, and their combination. To test this, we exposed *F. arundinacea* to simulated cool-season drought (60% rainfall reduction) and year-round warming (ambient +3 °C) using a factorial field manipulation experiment, PAstures and Climate Extremes (PACE), at Western Sydney University, Australia. We hypothesized the following. (H1) Both drought and warming reduce plant production (above- and belowground) and standing root biomass while increasing the relative allocation of biomass belowground (*f*_BNPP_ and RMF). (H2) Fine root traits exhibit plasticity in response to drought and warming, with increasing SRL and decreasing RTD and mean root diameter, reflecting a shift towards more acquisitive trait values that may reduce the effects of climate extremes by promoting soil resource acquisition. (H3) Drought increases and warming decreases belowground storage (NSCs and N), with consequences for post-drought recovery of aboveground productivity. During drought, the senescence of shoots and reallocation of C and N reserves to crowns and roots is expected; however, higher maintenance costs with warming are expected to reduce belowground C storage.

## Materials and methods

### Experimental site

This study was conducted from the austral winter in June 2019 to the autumn of March 2020 at the PACE (PAstures and Climate Extremes) field facility on the Hawkesbury campus of Western Sydney University in Richmond, New South Wales, Australia (S33.60972, E150.73833, elevation 25 m asl). This site receives a mean annual rainfall of 806 ± 37 (SE) mm with a large inter-annual variability ranging from 500 mm to 1400 mm over the past 30 years (Australian Government Bureau of Meteorology, Richmond—UWS Hawkesbury Station 1980–2010). Summer (December–February) is typically the wettest season, and winter (June–August) is generally the driest. Climate models predict a decrease in cool-season (winter and spring) rainfall under most scenarios, along with an increase in the frequency and severity of drought throughout south-eastern Australia (CSIRO, 2020). The mean annual temperature is 17.2 °C, with seasonal daily mean maximum/minimum temperatures of 29.4/18.8 °C in summer and 17.3/3.2 °C in winter. However, this region is predicted to experience +2.5 °C to 4 °C warming by the end of this century (Pearce *et al.*, 2007; Sherwood *et al.*, 2020; IPCC, 2021). The soil is a Blackendon Sand, with a sandy loam texture (sand, 81%; silt, 6%; clay. 11%), a water holding capacity of 20–22%, soil organic matter content of ~1.8%, and a pH of ~5.7 in the top 15 cm.

### Experimental design and treatments

The PACE experimental facility consists of six polytunnel shelters constructed from galvanized steel frames and covered with a single layer of polyethylene 180 μm plastic (Argosee greenhouse technology Pty Ltd, Australia) to intercept all ambient rainfall. Each shelter is 48 m long × 8 m wide × 4.6 m in height, oriented along a SW–NE axis with the open ends facing the direction of the prevailing wind. In addition, the long sides of the shelter are open to a height of 1.5 m to allow free airflow that minimizes shelter effects on microclimate. Each shelter has eight plots (4 m × 4 m) that are further subdivided into four subplots (2 m × 2 m each) with different pasture species growing at the subplot level. In each shelter, plots were assigned to drought and warming treatments in a factorial design, which allowed us to evaluate interactions between co-occurring climate treatments and root trait plasticity responses to these treatments by comparing them with control. Plots were established during 2017–2018 from seed. This study reports data from the 2019 cool-season drought and the following recovery period. Before sward establishment, the surface soil in each shelter was rotary tilled to a depth of 12 cm to homogenize the upper soil profile. Root barriers (Vercan™, textured high-density polyethylene, 1 mm in thickness; Argosee Greenhouse Technology Pty Ltd, Australia) were installed to a depth of 90 cm around all 4 × 4 m plots to ensure hydrological isolation between treatments. The 2 × 2 m subplots were separated by a 30 cm deep root barrier (Vercan™, 0.7 mm in thickness) to minimize root intrusion among pasture types. Furthermore, there was a buffer area of 1 m between each plot and 0.5 m between subplots, which included pavers aboveground to enable plot access.

This study focused on the perennial C_3_ pasture grass *F. arundinacea* (Quantum II MaxP cultivar). *Festuca arundinacea* is native to large regions of Europe, temperate Asia, and North Africa (USDA-NRCS, 2016) and is naturalized in most temperate regions worldwide. It is widely planted for use as pasture, hay, and silage (Gibson and Newman, 2001). The species is known for its resilience and vigour compared with other grass species (Preston and Hill, 1997). The optimum growth temperature for this cultivar ranges from 15 °C to 30 °C (Hill *et al.*, 1985). However, the ongoing and predicted changes in climate, rainfall reduction and atmospheric warming are expected to negatively affect *F. arundinacea* production in many of its growing regions, with substantial consequences for the pasture industry (Cullen *et al.*, 2009; McCulley *et al.*, 2014; Norton *et al.*, 2016; Godde *et al.*, 2021). Here, we exposed *F. arundinacea* to drought and warming, in factorial combination, to determine adaptability to future climate, specifically in relation to belowground traits.

The drought treatment comprised a 60% reduction in irrigation amount relative to control, applied during the 6 month austral winter and spring period (cool-season;  June through November). The following 6 month summer and autumn period represents the post-drought recovery period when all plots received the control irrigation amount. The 60% reduction in the amount of cool-season rainfall represents the upper end of climate model drought predictions for south-eastern Australia at the end of the century under RCP8.5 (CSIRO, 2020). We collected historical rainfall records from the last 128 years from the Australian Bureau of Meteorology for Richmond, NSW (station 067105, 5 km away) to generate the daily probability distribution for rainfall to derive the control irrigation schedule. Control plots received the scheduled amount, but there was a 60% reduction in the size of each rainfall event for the drought treatment (from 1 June to 30 November 2019) (Supplementary Fig. S1A).

The warming treatment (elevated temperature, eT) comprised a year-round +3 °C increase in canopy temperature relative to ambient temperature (aT). Warming was achieved using a heating array that included eight 1000 W ceramic heaters (FTE 1000W, Ceramicx, Ireland) installed 1.4 m above the ground surface in each heated 4 × 4 m plot (Kimball *et al.*, 2008). The heaters were angled to give uniform coverage of infrared (IR) radiation across the four composite subplots. The power level of the heaters was adjusted each minute via pulse width modulation using a solid-state relay controlled by a data logger (CR1000, Campbell Scientific) with a proportional-integral control algorithm. Target temperatures for these plots were controlled via feedback from IR sensors (SI-100, Apogee Instruments, Logan, UT, USA) mounted at 3.8 m height, recording canopy surface temperatures every 5 min. The heating treatment maintained daily temperature fluctuations in real-time by warming the eT plots +3 °C more than the recorded temperature from aT plots from the paired plots within each water treatment, namely aT–control (C) paired to eT–C and aT–drought (D) paired to eT–D (data shown in Supplementary Fig. S1C–H). Experimental warming using IR radiation may result in a small reduction in air relative humidity (~1%) within the plant canopy (LeCain *et al.*, 2015), as IR radiation heats plant and soil surfaces and not air. Thus, we monitored only canopy and soil surface temperatures.

### Environmental monitoring

Each shelter had data loggers which recorded the real-time environmental conditions under each climate treatment. Time Domain Reflectometers (CS616, Campbell Scientific) and soil temperature probes (T107; Campbell Scientific) recorded soil volumetric water content (0–15 cm depth) and soil temperature (at 0–5 cm), respectively, at 15 min intervals. Air temperature (Series RHP-2O3B) and relative humidity (Dwyer Instruments Inc., USA) sensors mounted in force-ventilated radiation shields were installed at 0.6 m height inside and outside shelters to record air temperature and relative humidity, respectively, every 5 min. Quantum sensors (Apogee quantum sensor, USA) installed inside (3 m height) and outside (6 m height) the shelter recorded photosynthetically active radiation (PAR) at 5 min intervals.

### Aboveground net primary production

During the 2019 drought period, all subplots were regularly harvested 5 cm above the soil surface using a sickle mower and hand shears, oven-dried (at 70 °C for 3 d), and weighed. The timing of harvests was based on grazing recommendations from the pasture industry, harvesting at the peak of vegetative growth at ~30 cm height (Clements *et al.*, 2003). Despite differences in rates of vegetative growth between control and treatment plots, all swards were harvested at a similar time, with timing referenced to control plots. The cumulative biomass of the three harvests conducted during the 6 month cool-season drought period was used to represent the aboveground net primary production (ANPP; g m^–2^ per 6 months). The harvest method mimics the cell-grazing technique, a form of rotational grazing. During cell-grazing, small paddocks are intensively grazed, followed by a long recovery period (MLA, 2020). The small plot size prevented us from using animals for grazing. Instead, we simulated the cell-grazing using the cut-and-carry method.

Aboveground biomass recovery following the 6 month drought (which ended on 30 November 2019) was measured in mid-March 2020. Post-drought aboveground vegetation was harvested 5 cm above the soil surface, in the same way as harvests during the cool-season drought period. This harvest captured 3.5 months of growth during the summer, where all plots received control precipitation amounts under both warming and ambient temperature. We excluded data from one of the six replicate shelters from aboveground biomass recovery analyses due to the destruction of this shelter during a severe storm at the end of November 2019.

### Belowground net primary production

BNPP was measured during the 6 month cool-season drought using open ingrowth cores (Li *et al.*, 2013). In brief, at the beginning of the 2019 drought period (in June), four randomly located holes (5 cm diameter and 20 cm deep) were excavated in each 2 × 2 m subplot and filled with root-free soil (local soil and sand mixture 3:1) and compacted to approximate the original soil bulk density. PVC collars of 5 cm diameter (2 cm depth) were used to mark these locations. At the end of the drought period in November 2019, roots from each marked location were sampled at two depths (0–10 cm and 10–20 cm) using a 5 cm diameter soil auger. Soil samples were sieved with a 2 mm sieve, and root material was washed to remove attached soil particles. Root material from the four cores was pooled into a single composite sample for each subplot, from which a subsample was scanned to measure root morphological traits (see below) and later oven-dried (at 70 °C for 3 d). The remaining root material was oven-dried before measuring chemical traits. Oven-dry mass of the composite root sample was used to determine root production on a ground area basis (g m^–2^ per 6 months) for each soil depth. However, our open ingrowth cores did not capture root crowns due to their small size. Here we show belowground production data for 0–20 cm after summing the two depths. We chose to sample roots at a depth of 0–20 cm since the majority (>80%) of root biomass occurred at this depth in our study.

### Standing root biomass and crown sampling

Standing root biomass was sampled at the end of the 6 month cool-season drought period, in November 2019, at two depths (0–10 cm and 10–20 cm) using a 5cm diameter soil auger; here we present summed data for 0–20 cm depth. Four randomly located soil cores were collected from each replicate plot and pooled to make a composite sample. Root samples were processed as described above. At the same time, root crowns were sampled in a 30 cm × 30 cm area in a similar position in all *F. arundinacea* subplots. Crowns were trimmed to remove aboveground vegetation, and root material was washed to remove attached soil particles before being oven-dried (at 70 °C for 3 d); root and crown data are presented separately.

### *f*_BNPP_ and RMF

The *f*_BNPP_ was calculated as the ratio of belowground net primary production to total plant net primary production.


$$\begin{array}{l} f_{BNPP}=\frac{BNPP}{ANPP+BNPP} \end{array}$$

The RMF was calculated as the fraction of plant biomass allocated to roots.


$$\begin{array}{l} RMF=\frac{Standing\;\;\;root\;\;\;biomass}{Aboveground\;\;\;biomass+Standing\;\;\;root\;\;\;biomass} \end{array}$$

*f*_BNPP_ and RMF do not include crown biomass.

### Morphological root traits

Washed fine root samples from ingrowth cores were scanned using an Epson Perfection 4990 scanner at 800 DPI (horizontal and vertical resolution) within 24 h of sampling. Scanned images were analysed using Winrhizo™ software (Regent Instrument Inc., Sainte-Foy, QC, Canada) to determine root length, diameter and volume. Scanned root samples were oven-dried (at 70 °C for 3 d) and weighed. SRL (m g^–1^) was calculated as the ratio of root length to root dry mass, and RTD (g cm^–3^) as the root dry mass to the root volume ratio.

### Root and crown chemical analysis

Tissue concentrations of NSCs (non-structural carbohydrates; soluble sugars and starch) in plant roots produced during the cool-season drought and in crowns sampled at the end of drought were measured using methanol:chloroform:water extraction and a colorimetric phenol–sulfuric acid assay (Tissue and Wright, 1995). In brief, oven-dried root or crown materials were ground into a fine powder using a ball mill (Retsch MM 400). Approximately 15 mg of ground material was extracted three times with 2 ml of methanol:chloroform:water (12:5:3 v/v/v) solution to separate the soluble sugars from the pellet fraction containing starch. The dried pellet containing the starch fraction was then treated with 5 ml of 35% perchloric acid for 30 min to hydrolyse the starch. Soluble sugar and starch concentrations were determined colorimetrically using the phenol–sulfuric acid method. Total NSC concentrations were calculated as the sum of soluble sugars and starch (Aspinwall *et al.*, 2016). N concentrations (%) were measured for root and crown samples using a CHN analyser (LECO TruSpec, LECO Corporation, St Joseph, MI, USA). NSCs can represent a significant but variable proportion of plant tissue mass and can thereby influence apparent values of tissue N concentrations. To account for this variability, we calculated NSC-free N concentrations using the formula below (Aspinwall *et al.*, 2016):


$$\begin{array}{l} N_{\left[NSC\text{-}free\right]}=\frac{N}{1-NSC} \end{array}$$

### Statistical analysis

All biomass and productivity data were scaled to dry mass per unit ground area (g m^–2^). All statistical analyses were performed using R 4.2.2 (R Core Team, 2022). We compared data from drought and warming treatments across shelters using a linear-mixed effects model (‘lme4’ package, Bates *et al.*, 2015), with drought and warming treatments and their interaction as fixed factors and shelters as a random factor to account for spatial blocking in the field. We obtained *P*-values of all fixed factors in a model using the Kenward–Roger approximation of the degrees of freedom (Halekoh and Højsgaard, 2014) with the ANOVA (test=‘*F*’) function from the ‘car’ package (Fox and Weisberg, 2019). Pairwise comparisons among treatment combinations (i.e. aT–C, aT–D, eT–C, and eT–D) were conducted using the R package ‘emmeans’ (Lenth *et al.*, 2021). Whenever required, we used natural logarithm or square root transformation to meet assumptions of normality, as reported in the footnotes of Table 1. To account for the zero-inflated distribution of recovery biomass, we used a non-parametric Wilcoxon signed rank test. However, the results from these non-parametric tests were similar to the linear mixed effect models, and the results from the Wilcoxon signed rank test are reported in Supplementary Table S1. Standardized major axis tests and routines were conducted to test bivariate relationships among root traits using the SMATR package in R (Warton *et al.*, 2012). In standardized major axis tests and routines, a single line describes the bivariate relationship regardless of which variable is *X* and which is *Y*. Standard least squares regression analysis was used to determine predictive relationships for ANPP and BNPP (dependent variable) in relation to belowground traits.

**Table 1:** Summary statistics for the effects of drought, warming, and their interaction on belowground biomass, productivity, and traits from a linear mixed effect model

Response variables	Drought		Warming		Drought×Warming	
	*F*	*P*	*F*	*P*	*F*	*P*
ANPP (g m^–2^ per 6 months)^*a*^	**28.6**	**<0.001**	**7.98**	**0.01**	1.18	0.29
BNPP (g m^–2^ per 6 months)	**61.3**	**<0.001**	**27.9**	**<0.001**	1.48	0.24
Standing root biomass (g m^–2^)^*b*^	**5.19**	**0.03**	**17.7**	**<0.001**	0.03	0.85
Crown biomass (g m^–2^)^*a*^	**20.43**	**<0.001**	**14.04**	**0.001**	1.96	0.18
Aboveground recovery (g m^–2^)	0.9	0.36	2.56	0.13	0.04	0.84
*f*_BNPP_	**16.23**	**0.001**	**10.8**	**0.004**	0.21	0.65
Root mass fraction	1.62	**0.22**	**6.18**	**0.02**	0.21	0.65
Specific root length (m g^–1^)	**4.7**	**0.04**	*4.04*	*0.06*	**4.64**	**0.04**
Mean root diameter (mm)^*a*^	**8.75**	**0.009**	**5.39**	**0.03**	*3.4*	*0.08*
Root tissue density (g cm^–3^)	0.04	0.84	0.25	0.62	0.02	0.88
Root soluble sugars (mg g^–1^)	3.06	0.1	0.26	0.61	0.001	0.97
Root starch (mg g^–1^)^*a*^	1.41	0.2	**8.11**	**0.01**	**9.02**	**0.008**
Root nitrogen (%)^*a*^	**147.2**	**<0.001**	**68.6**	**<0.001**	**9.78**	**0.006**
Crown soluble sugars (mg g^–1^)	0.94	0.3	0.73	0.4	0.002	0.9
Crown starch (mg g^–1^)	**5.38**	**0.03**	**3.53**	**0.07**	3.05	0.1
Crown nitrogen (%)	**7.45**	**0.01**	**15.57**	**0.001**	0.04	0.8

Bold font represents statistical significance (*P*<0.05), and italics represent effects at the *P*<0.1 level of significance. Superscripts *a* and *b* indicate responses that were natural logarithm and square root transformed, respectively, to meet assumptions of normality.

## Results

### Plant production and biomass

**Fig. 1.**
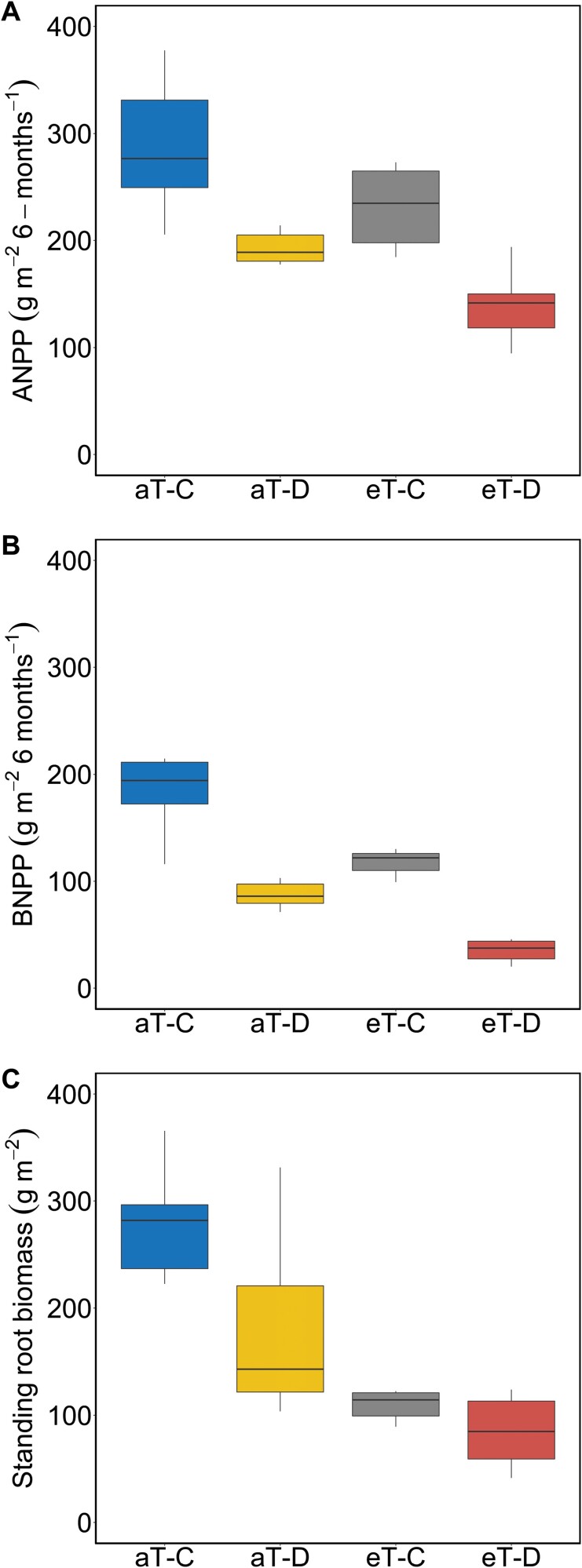
(A) Aboveground net primary production (ANPP), (B) belowground net primary production (BNPP), and (C) standing root biomass to 20 cm depth in *F. arundinacea* in response to factorial cool-season drought and year-round warming treatments. Abbreviations aT–C (ambient temperature–control), aT–D (ambient temperature–drought), eT–C (elevated temperature–control), and eT–D (elevated temperature–drought) represent treatment combinations (*n*=6 plots). Horizontal lines within boxes indicate medians, and the upper and lower edges of the box plots represent the 25th and 75th percentiles. The top bar shows the maximum value, and the bottom bar the minimum value. Statistics for these figures are shown in Table 1. The ANPP data shown in (A) have been reported previously (Churchill *et al.*, 2022) and are reproduced here for convenience.

During the cool-season, ANPP was reduced by drought (–30%), warming (–9%), and their combination (–51%), relative to control (Fig. 1A). Similarly, BNPP was reduced by drought (–53%), warming (–35%) and the combination of both treatments (–77%) (Fig. 1B). Both drought and warming also significantly reduced standing root biomass (Fig. 1C). However, there were no interactions between drought and warming for ANPP, BNPP, or standing root biomass (Table 1).

### Belowground biomass allocation

**Fig. 2.**
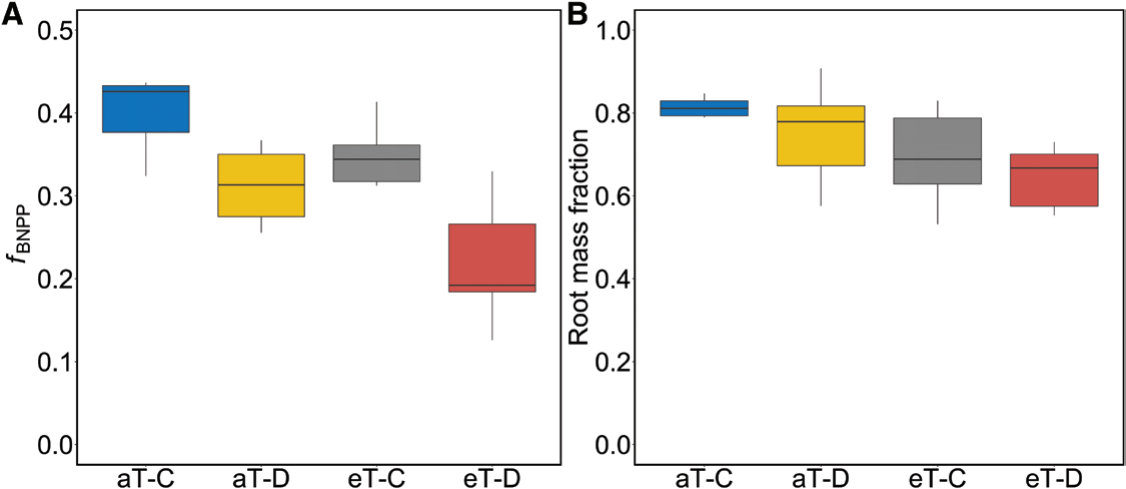
(A) The fraction of net primary production allocated belowground (*f*_BNPP_) and (B) root mass fraction (n=6 plots) in *F. arundinacea* in response to factorial drought and warming treatments. Statistics for these figures are shown in Table 1, and all abbreviations in the figure match those in Fig. 1.

Drought and warming significantly reduced *f*_BNPP_ although there were no significant interactions between treatments (Fig. 2A; Table 1). In contrast, only warming reduced RMF (Fig. 2B; Table 1).

### Root morphological traits and their plasticity

**Fig. 3.**
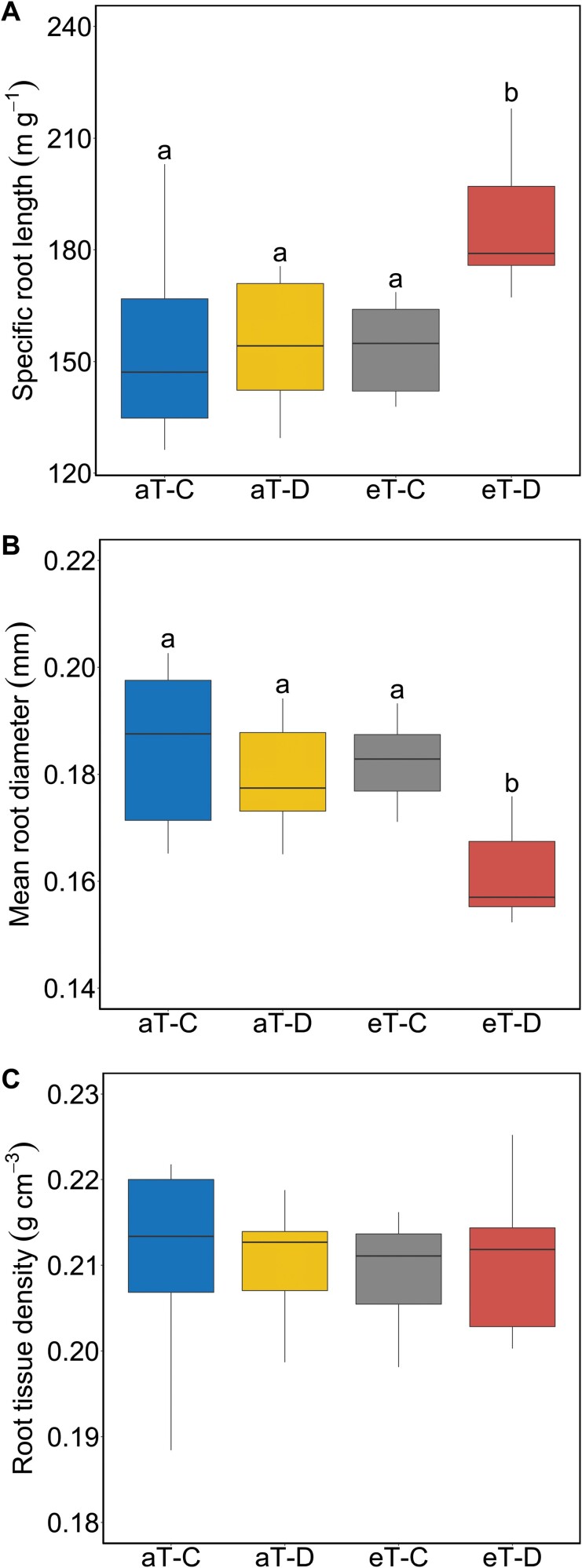
Plasticity in the fine root morphological traits, (A) specific root length, (B) mean diameter, and (C) tissue density in *F. arundinacea* roots produced during the 6 month cool-season in 2019, in response to factorial drought and warming treatments. Statistics for these figures are shown in Table 1. Different letters above the boxes indicate a post-hoc pairwise comparison between treatments (*n*=6 plots) when the interactions between climate treatments are significant. All abbreviations in the figure match those in Fig. 1.

There was a significant interaction between drought and warming for SRL and mean root diameter (Table 1). Drought treatment significantly increased SRL (Fig. 3A) and decreased mean root diameter (Fig. 3B), but only under elevated temperature. RTD was unaffected by either drought or warming (Fig. 3C; Table 1).

### Root and crown chemical traits

**Fig. 4.**
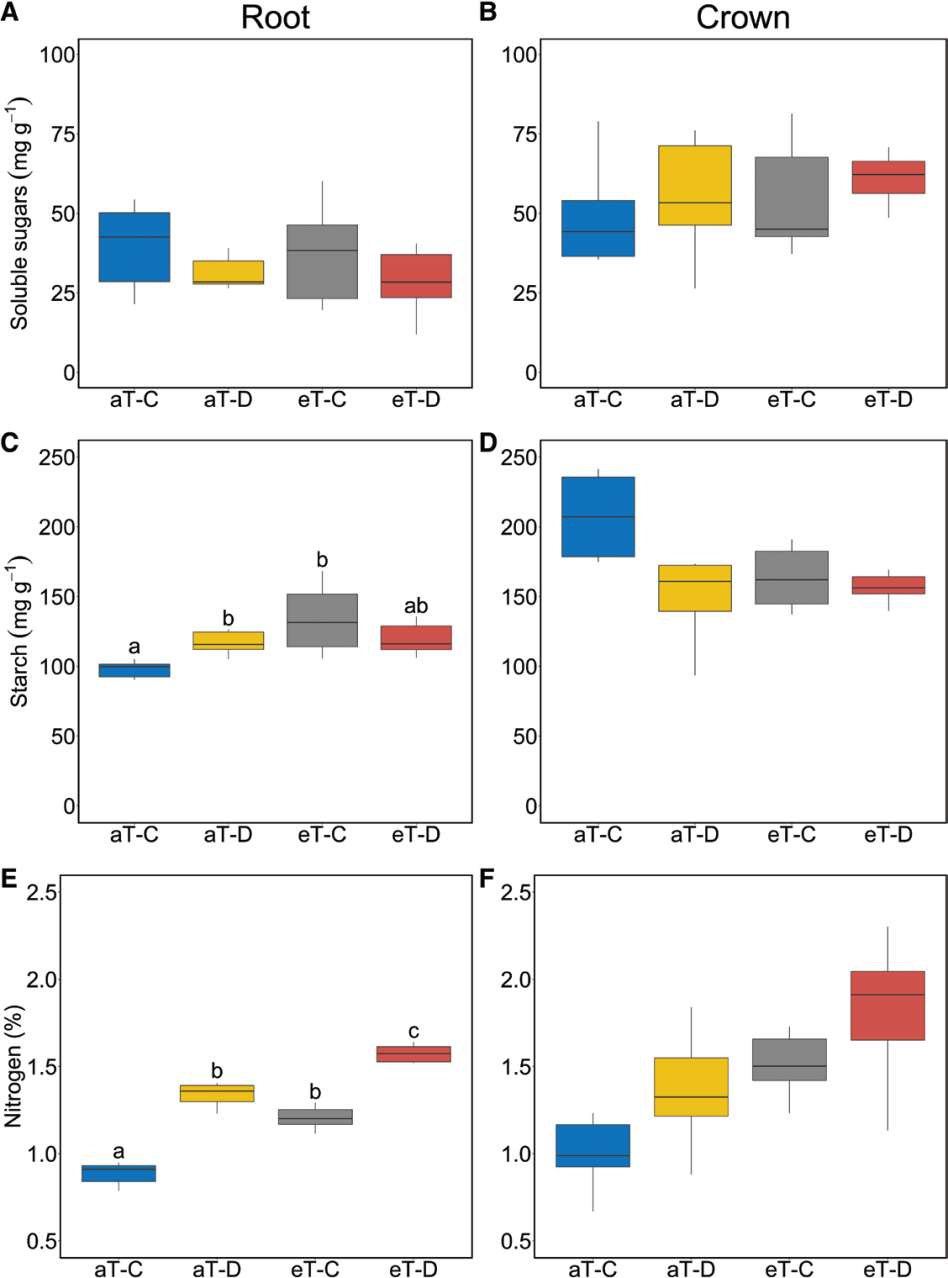
Plasticity in belowground chemical traits, soluble sugars in (A) root and (B) crown, starch in (C) root and (D) crown, and nitrogen concentrations in (E) root and (F) crown in *F. arundinacea* in response to factorial drought and warming treatments. Different letters above the boxes indicate a post-hoc pairwise comparison between treatments (*n*=6 plots) when the interactions between climate treatments are significant. Root chemical traits were measured in fine roots produced during the 6 month cool-season in 2019 and crown traits from samples collected at the end of the 2019 drought treatment period. All abbreviations in the figure match those in Fig. 1.

Neither drought nor warming affected soluble sugar concentrations in roots or crowns (Fig. 4A, B; Table 1). Relative to control plots, root starch concentrations were significantly higher in drought (aT–D, +28%) and warming (eT–C, +43%) treatments, but their combined effect was less than additive (eT–D, +27%) (Fig. 4C; Table 1). In contrast, both drought and warming decreased crown starch concentrations (Fig. 4D; Table 1) but increased root and crown N concentrations (Fig. 4E, F; Table 1).

### Correlations among belowground traits

**Fig. 5.**
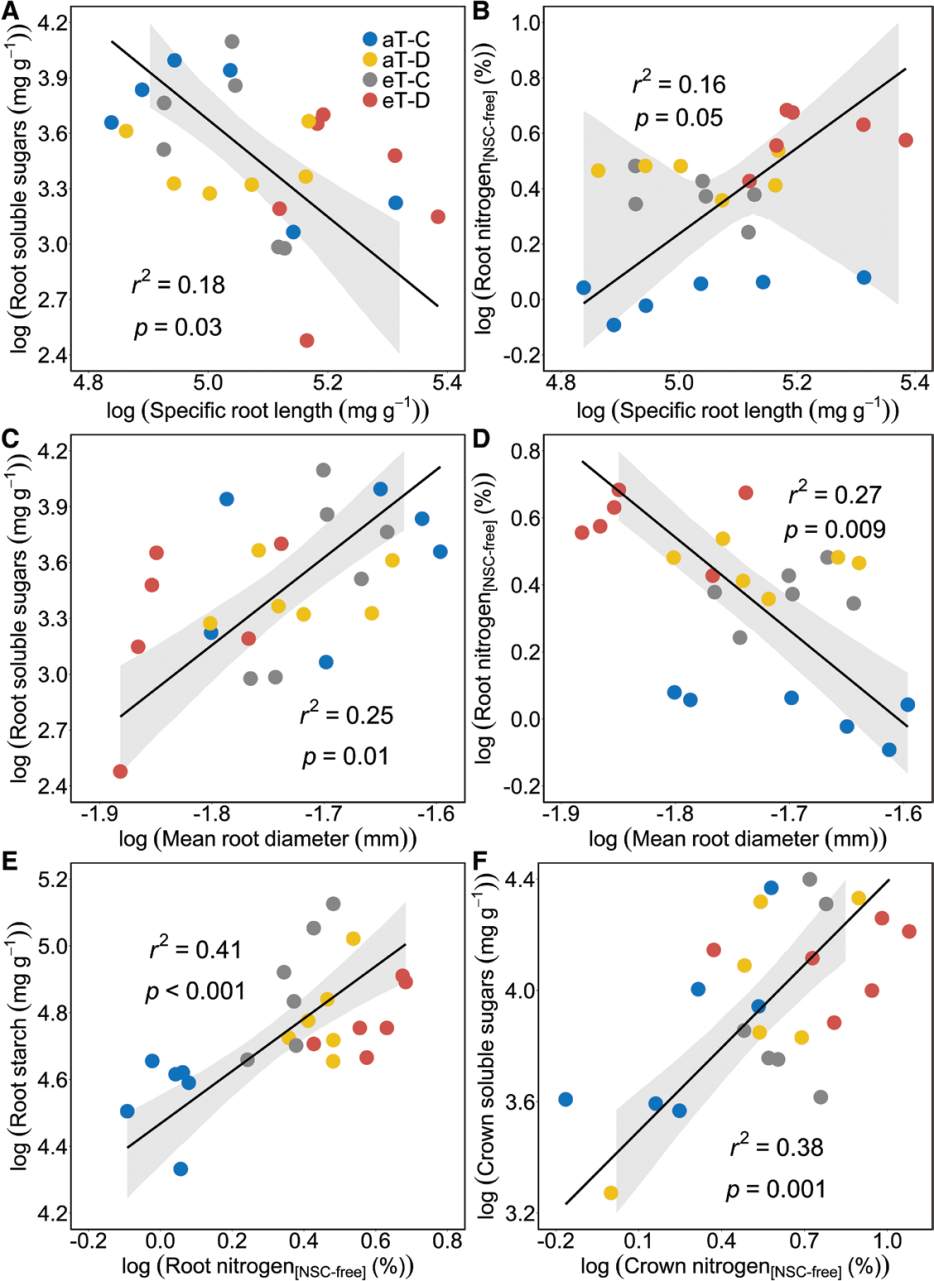
Correlations among root traits in *F. arundinacea* during factorial drought and warming treatments examined using standardized major axis tests and routines. A solid line with 95% confidence intervals represents significant trait correlations. Both the *x*- and *y*-axes are log (natural log) transformed. All abbreviations in the figure match those in Fig. 1.

Belowground traits were strongly correlated, such that root soluble sugar concentrations decreased, and root N concentrations increased with increases in SRL (Fig. 5A, B). In contrast, root soluble sugar concentrations were positively, and root N concentrations negatively, related to mean root diameter (Fig. 5C, D). There were positive correlations between N and NSC concentrations in belowground tissues, such that root starch concentrations increased with increasing root N_[NSC-free]_ concentrations. Similarly, crown soluble sugar concentrations increased with crown N_[NSC-free]_ concentrations (Fig. 5E, F).

### Plant aboveground productivity responses in relation to belowground traits

**Fig. 6.**
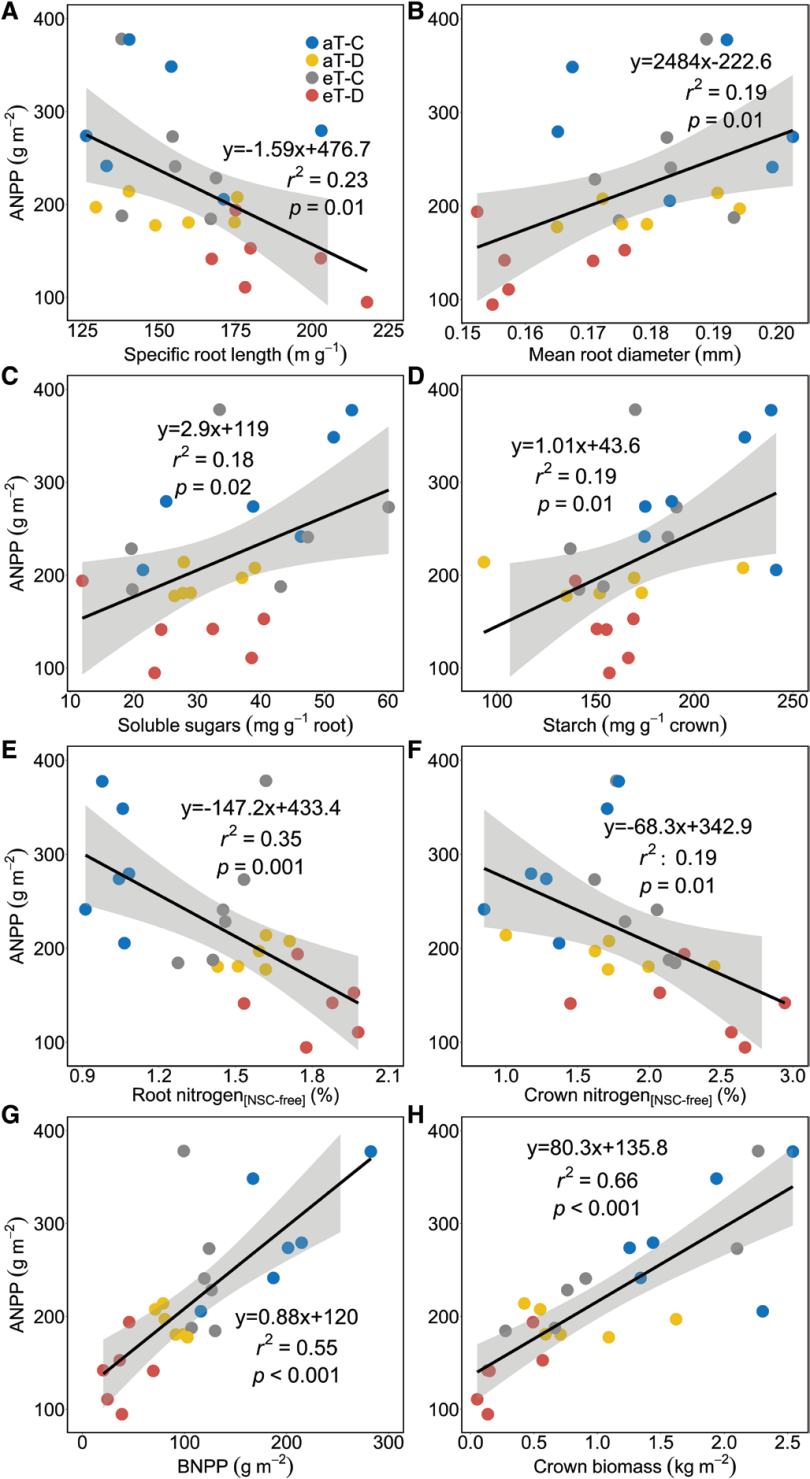
Aboveground net primary production (ANPP) during the 6 month cool-season in *F. arundinacea* in relation to belowground traits during factorial drought and warming treatments. A solid line with the standard error represents significant linear relationships. All abbreviations in the figure match those in Fig. 1.

Standard least squares regressions demonstrated linear relationships between aboveground production and belowground traits in *F. arundinacea*. During drought, warming, and their combination, ANPP was positively associated with mean root diameter (Fig. 6B), root soluble sugar (Fig. 6C) and crown starch concentrations (Fig. 6D). Additionally, the negative relationships between ANPP and SRL (Fig. 6A), and root and crown N concentrations (Fig. 6E, F) demonstrate reductions in plant production with increases in those traits. The positive relationships between ANPP and BNPP (Fig. 6G) and crown biomass (Fig. 6H) also explain some of the variation in plant production.

### Belowground production responses in relation to root traits

**Fig. 7.**
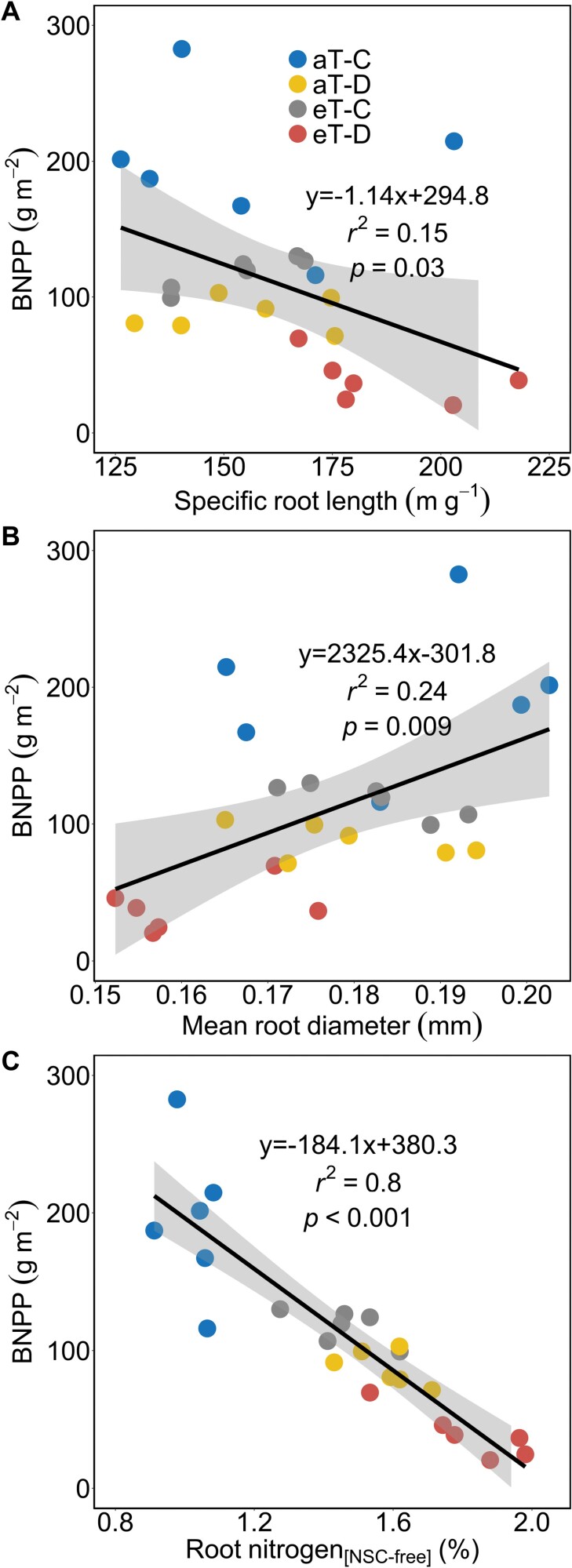
Belowground net primary production (BNPP) during the 6 month cool-season in *F. arundinacea* in relation to root traits during factorial drought and warming treatments. A solid line with the standard error represents significant linear relationships. All abbreviations in the figure match those in Fig. 1.

Standard least squares regressions demonstrated linear relationships between BNPP and root traits. BNPP declined with increasing SRL (Fig. 7A) and root N concentrations (Fig. 7C) in response to drought, warming and their combination. In contrast, BNPP increased with increasing mean root diameter (Fig. 7B).

### Crown biomass and post-drought aboveground biomass recovery

**Fig. 8.**
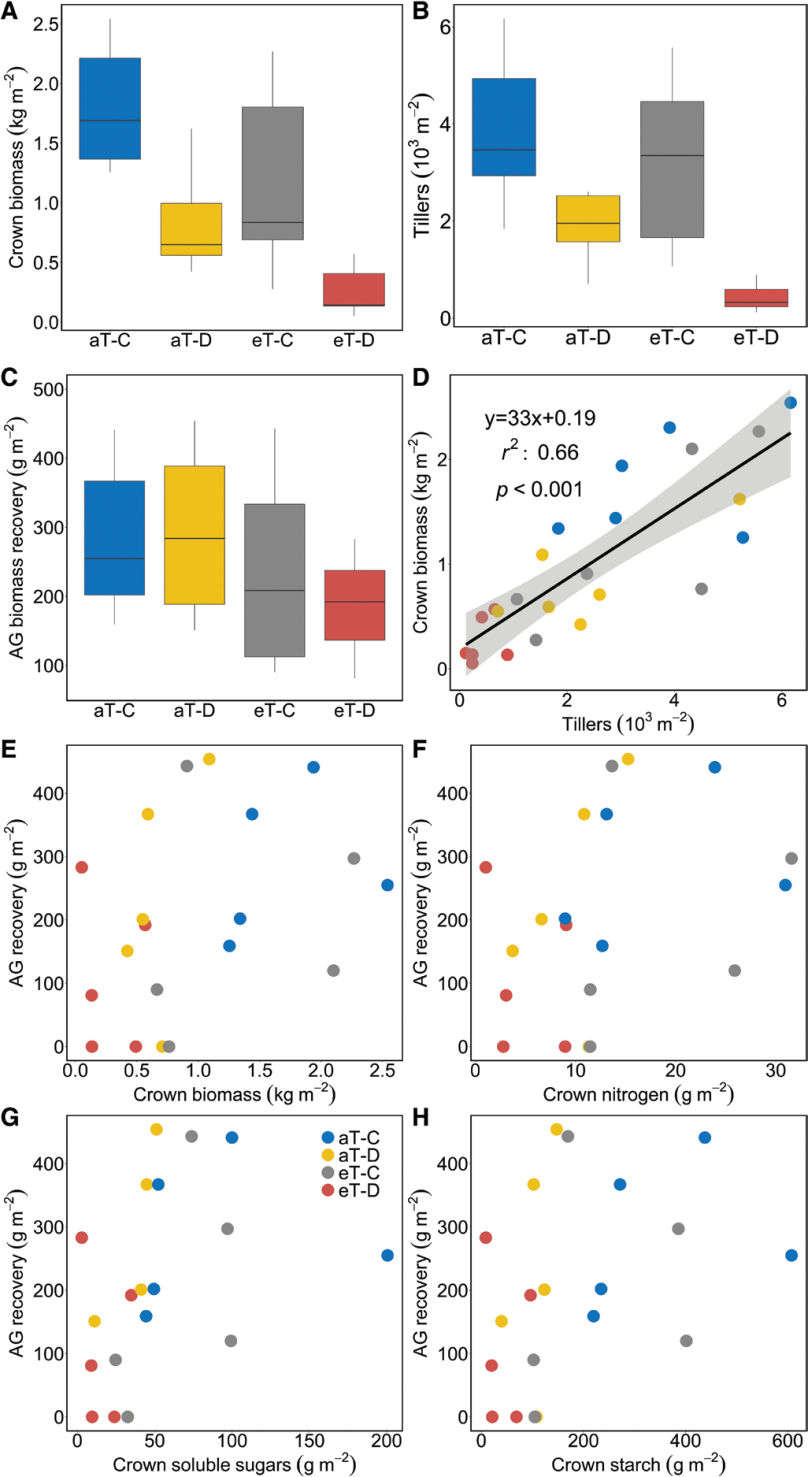
(A) Crown biomass, (B) tiller density, and (D) bivariate relationships between crown biomass and tiller density in *F. arundinacea* in response to factorial drought and warming treatments. (C) Aboveground biomass following the cool-season drought in 2019. Post-drought aboveground biomass in relation to (E) crown biomass and crown storage of (F) nitrogen, (G) soluble sugar, and (H) starch. A solid line with the standard error in (D) represents significant linear relationships. Statistics for these figures are shown in Table 1, and all abbreviations in the figure match those in Fig. 1.

Drought and warming significantly reduced crown biomass at the plot level, with the biggest reductions seen in plots exposed to the combined treatment (–85% relative to ambient control) (Fig. 8A; Table 1). Drought also reduced tiller density at the plot level under both ambient (–41% compared with aT–C) and elevated temperatures (–76% relative to eT–C, Fig. 8B). Standard least squares regression shows a positive linear relationship between crown biomass and tiller density at the end of the cool-season drought (*r*^2^=0.68, *P*<0.001, Fig. 8D). The aboveground biomass recovery following the 2019 cool-season drought was similar among treatments (Fig. 8C). However, neither the plot-level crown biomass nor crown storage of N or NSCs explained the post-drought recovery of aboveground biomass (Fig. 8E–H).

## Discussion

Here, we addressed a knowledge gap relating to belowground trait plasticity responses to future warmer, drier climates and their relationships to aboveground production using a factorial field experiment. The effects of drought and warming on above- and belowground biomass production and the fraction of biomass allocated to belowground were negative, independent, and thus additive. However, the combined effects of drought and warming resulted in a shift in root trait values towards more acquisitive combinations, notably higher SRL, lower mean diameter and higher N concentrations. Greater mean root diameter and higher root soluble sugar and crown starch concentrations were predictors of plant aboveground production during drought and warming. However, in contrast to our expectations, there were no relationships between post-drought aboveground biomass and pre-existing belowground NSC and N reserves.

### Plant production and biomass allocation during drought

Drought reduced cool-season plant production, both above- and belowground, demonstrating drought sensitivity of *F. arundinacea*. However, the greater drought sensitivity of BNPP relative to ANPP observed in our study supports findings from grassland studies elsewhere (Frank, 2007; Carroll *et al.*, 2021), although there have been contrasting responses observed for *f*_BNPP_ and RMF. This finding is at odds with expectations based on the functional equilibrium theory (Lambers, 1983), whereby plants are expected to increase *f*_BNPP_ (Hui and Jackson, 2006; Franco *et al.*, 2020) and RMF (Poorter *et al.*, 2012; Eziz *et al.*, 2017) in response to drought since a greater proportional biomass investment belowground can increase the acquisition of water and other soil resources (Wilcox *et al.*, 2015; Zhang *et al.*, 2017; Chandregowda *et al.*, 2022a). The observed reduction in BNPP and *f*_BNPP_ in our study might be due to the large drought effect size on aboveground physiology, such as a reduction in leaf water potential and associated impacts on overall plant C assimilation (reported in Jacob *et al.*, 2022) and the resulting decrease in the availability of carbohydrates to invest in belowground production. A reduction in new C allocation belowground during drought observed in pulse-labelling studies elsewhere (Hasibeder *et al.*, 2015; R.Wang *et al.*, 2021) supports our findings. On another note, the stimulatory effect of grazing or mowing is expected to increase BNPP and *f*_BNPP_ by facilitating increased availability of light, water and nutrients (McNaughton, 1976; Pandey and Singh, 1992; Loeser *et al.*, 2004; Xu *et al.*, 2012). However, aboveground regrowth may have been constrained during drought due to the limitation of soil moisture for root growth, as suggested by Frank (2007). Since all plots in this study were harvested regularly, in line with grazing offtake recommendations (Clements *et al.*, 2003), we were unable to directly evaluate the effects of drought and clipping on belowground biomass allocation.

### Plant production and belowground biomass allocation during warming

Despite model projections suggesting widespread positive plant responses to warming during the winter months, results are inconsistent across study areas, seasons, and species (Bloor *et al.*, 2010; Ryan *et al.*, 2017). Here, we observed a reduction in *F. arundinacea* productivity in response to +3 °C warming, in contrast to previous reports of beneficial effects of warming on grassland productivity (Gao *et al.*, 2016), especially during cool-season months in higher latitude ecosystems (Naudts *et al.*, 2011; Mori *et al.*, 2014). In contrast, but in support of our findings, a study by Wu *et al.* (2021) showed a consistent reduction in ANPP over 30 years in response to observed climate warming across all major grassland types, including temperate grasslands in northern China, where soil temperature was the key factor limiting plant production. In addition, De Boeck *et al.* (2008) also measured a reduction in ANPP and BNPP in their grassland warming experiment, which was associated with a lowering of soil water content. Based on earlier findings, we believe that the negative effects of warming on plant production recorded in our study might be associated with the exceedance of species’ thermal optima (Drake et al., 2015, 2017) or higher soil temperature (Wu *et al.*, 2021), and/or warming-induced soil drying (Sherry *et al.*, 2008; Bowman *et al.*, 2014). Interestingly, soil volumetric water content in warmed plots (eT–C) increased with declining productivity in our study, indicating a reduction in plant water uptake (Supplementary Fig. S1B), which is in line with observations from a similar warming experiment in annual-dominated California grassland at the Jasper Ridge Biological Preserve, Stanford, USA (Zavaleta *et al.*, 2003). Jacob *et al.* (2020) report that the photosynthetic thermal optimum for *F. arundinacea* varied between 28 °C and 29 °C in their glasshouse study. However, springtime air temperatures in our study were regularly higher than this, and it is likely, therefore, that exceedance of *F. arundinacea*’s photosynthetic thermal optimum contributed to the observed reduction in plant productivity.

In addition to the reduction in ANPP and BNPP with warming, the fraction of primary production allocated belowground was also lower under warming in contrast to our hypothesis (H1). The possible explanations for this response may be an overall reduction in C fixation under warming, and thus a decrease in available C to allocate belowground or increased C loss via belowground maintenance respiration (Wertin *et al.*, 2011; Jacob *et al.*, 2020). In contrast to our findings, higher belowground production relative to aboveground was observed in response to warming in the alpine tundra (Yang *et al.*, 2020).

### Interacting effects of warming and drought on plant biomass and production

While the effects of drought and warming have been studied independently in a large number of studies, their interactions have received far less attention, even though they often co-occur (Lobell *et al.*, 2015; Nankishore and Farrell, 2016). As expected, based on studies elsewhere (Cochrane *et al.*, 2015), the combined effects of drought and warming on ANPP, BNPP, and belowground biomass (standing root biomass and crown biomass) were negative, independent, and thus additive. Both drought and warming are known to impair plant physiological processes such as stomatal conductance and leaf cooling, which will potentially lead to heat stress, reducing CO_2_ exchange and causing senescence of aboveground plant parts (Boeck *et al.*, 2011). Furthermore, the co-occurrence of drought and heat exacerbates the effects of drought directly by increasing the atmospheric vapour pressure deficit and indirectly by speeding up the process of soil drying due to increased evapotranspiration (Yuan *et al.*, 2019; Grossiord *et al.*, 2020; Lian *et al.*, 2021; Yaffar *et al.*, 2021). Drought and warming treatments each affected soil water content, but responses differed through time, probably owing to changes in both inputs and treatment effects on plant demand (Supplementary Fig. S1B). Additionally, consistently higher soil temperature in the combined drought and warming treatment (Supplementary Fig. S1H) might have increased maintenance respiration belowground and contributed to the reduction in total plant production (Huang *et al.*, 2005; Chandregowda *et al.*, 2022b).

### Root traits during drought, warming, and their combined treatment

Plant roots show shifts in their trait values via phenotypic plasticity along the root economic spectrum, adjusting to changes in their growing environment. Interestingly, *F. arundinacea* root traits responded only to the combination of drought and warming by producing thin roots with higher SRL and lower mean root diameter in support of our hypothesis (H2). The observed root trait plasticity, such as an increase in SRL or reduction in mean root diameter, might be due to increased root branching or an increase in the production of first-order roots rich in N driven by lower SWC in the combined drought and warming treatment compared with the warmed (eT–C) plots (Supplementary Fig. S1B) (Ouimette *et al.*, 2013; McCormack *et al.*, 2015). In line with this, SRL was positively, and mean root diameter negatively, associated with root N concentrations, a proxy for higher root metabolic activity (Tjoelker *et al.*, 2005); this supports the idea that roots with a higher absorption area maintain higher rates of soil resource acquisition and metabolic activity under stressed environmental conditions (Bardgett *et al.*, 2014). We observed no significant root trait plasticity in response to warming (eT–C), contrary to global syntheses of observational data showing a positive effect of temperature on root diameter and a negative or neutral effect on SRL (Valverde-Barrantes *et al.*, 2017; Fort and Freschet, 2020). The lack of an increased demand for root water absorption due to adequate soil moisture in warming (eT–C) plots might be the reason for the lack of observed shifts in root traits in this treatment (Supplementary Fig. S1A, B).

Plasticity in C and N allocation belowground can help plants adjust to environmental stresses via maintaining water absorption in a drying soil and promoting recovery following disturbance (Chaves, 1991; Sanders and Arndt, 2012). Here, we found no change in root soluble sugar concentrations in response to either drought or warming. We did find an increase in root starch concentrations during drought, but the significant interaction between climate treatments suggests that the disappearance of the drought-associated increase in starch under high-temperature conditions may be associated with increased respiration rates. This supposition is supported by evidence from a controlled-environment study involving *F. arundinacea* (Chandregowda *et al.*, 2022b), which found higher root respiratory C loss in response to the combination of drought and warming, compared with drought and warming on their own. In addition, here we observed higher root N concentrations and a positive association between root N and root starch concentrations during climate stress which may be indicative of higher root metabolic activity (Tjoelker *et al.*, 2005; Jia *et al.*, 2013). The reduction in root soluble sugars combined with an increase in SRL also supports the idea of higher root respiratory maintenance under climate stresses.

### Relationships between root traits and plant production

Plant production responses to drought and heat stress are complex, involve a multitude of interacting traits and processes, and thus are difficult to infer using single trait studies. Bivariate regressions helped us decipher relationships between root traits and plant production. ANPP under climate treatments was associated negatively with SRL in this study. These findings differ from previous studies focused on water limitation, which have shown that the production of acquisitive roots with higher SRL helps plants to overcome water limitation due to their ability to explore more soil volume and extract water from smaller pores (Bardgett *et al.*, 2014; Freschet *et al.*, 2017; Chandregowda *et al.*, 2022a). However, plasticity in root traits in this study, for example increased SRL and lower mean root diameter, was evident only under the combined drought and warming treatments. In addition, the positive association between ANPP and mean root diameter may be explained by the greater capacity of thicker roots to store NSC reserves, thereby facilitating water absorption via osmosis and contributing to growth and tissue metabolic activity during periods of environmental stress (Morgan, 1984; Ozturk *et al.*, 2021). Alternatively, thicker roots with greater diameters can maintain plant production under unfavourable environmental conditions by facilitating increased mycorrhizal colonization and water uptake (Bergmann *et al.*, 2020; de la Riva *et al.*, 2021), although we did not evaluate mycorrhizal colonization in the current study. On another note, the observed negative relationship between thinner roots and ANPP is in the opposite direction to previous findings (Freschet *et al.*, 2021), suggesting an alternative hypothesis: that observed trait differences such as lower root diameter might have been the cause of delayed plant development under the drought and warming combination rather than adaptive plasticity. In support of this alternative hypothesis, there was a negative relationship between mean root diameter and root N concentrations, where root N concentrations were shown to decline with root age (Ceccon *et al.*, 2016).

The varied responses of BNPP to different treatment combinations in this study were also explained using linear regression relationships between root traits and belowground production. We observed a trade-off between root trait plasticity and BNPP, with a higher BNPP positively associated with mean root diameter but negatively with SRL and root N. Higher root length for absorption (higher SRL) and higher root N to support metabolic activity might reduce the requirement for C investment in belowground production. Again, the lower root diameter in plots with lower belowground production supports our alternative hypothesis that reduced plant development in the combined drought and warming treatment was the key reason for observed differences in root traits.

### Crown storage and post-drought recovery

The ability of grasses to recover following climatic stress is a function of their belowground C storage (Brunner *et al.*, 2015; Piper and Paula, 2020), with perennial grasses storing most of their C reserves in their root crown (White, 1973). Concentrations of NSCs such as starch and soluble sugars were higher in the crown relative to the root system, supporting the understanding that the crowns are the major belowground storage organ in perennial grasses. However, we found that climate treatments had a negative effect on plot-level crown biomass. This reduction in crown biomass was associated with lower numbers of tillers at the plot level. A higher C loss via respiration relative to C assimilation, along with the periodic biomass clipping that took place during the climate treatments, might have lowered tiller production or increased tiller mortality, as has been reported from a comparable controlled-environment study of drought and warming effects on *F. arundinacea* (Chandregowda *et al.*, 2022b).

Our finding of no change in soluble sugar concentrations in roots and crowns and a reduction in crown starch concentrations in response to drought and warming appears contrary to previous findings on the reallocation of C reserves to belowground organs under unfavourable environmental conditions (Munné-Bosch, 2014; Zeppel *et al.*, 2014; Pausas *et al.*, 2016; Lubbe *et al.*, 2021). Varied responses to climate stresses of different NSC pools in the crown could reflect differing patterns of utilization of stored reserves under extreme climatic conditions. For instance, in addition to the greater demand for stored belowground C during regrowth followed by periodic harvesting, the increased consumption of NSCs for tissue maintenance during climatic stresses might have accelerated the hydrolysis of starch into soluble sugars, resulting in the reduction of starch reserves. Hultine *et al.* (2021) demonstrated reductions in starch, but no changes in the soluble sugar concentrations in their episodic defoliation experiment in an invasive shrub. Alternatively, the probable reallocation of crown NSCs to roots to maintain root metabolic activity under extreme climate conditions might explain reductions in crown starch. However, we have no direct means of differentiating between C allocated to storage and that used for regrowth of shoots or roots.

An increase in crown N in response to climate treatments, a finding from this study, suggests that plants increase storage functions under environmental stresses. There was a positive relationship between crown N and crown soluble sugar concentrations under drought and warming, supporting the supposition of increased belowground storage in response to environmental stress. However, contrary to our hypothesis (H3), there were no relationships between post-drought aboveground biomass and plot-level crown biomass or crown NSC and N reserves. In addition, these findings were also in contrast to other studies, which have shown the importance of belowground C and N storage in post-drought recovery (Bell and Ojeda, 1999; Zeppel *et al.*, 2014; Pausas *et al.*, 2016). Reduction in plot-level crown biomass, crown starch storage, and reduced tiller production or increased tiller mortality under climate extremes might explain the lack of correlation between aboveground biomass recovery and crown N and NSC reserves, while subsequent crown mortality could also play a role.

### Conclusion

In conclusion, our findings highlight the sensitivity of *F. arundinacea* pasture production under a future warmer and drier climate, particularly particularly when these co-occur. Moreover, *F. arundinacea*’s wide habitat range may suggest comparable responses in other widespread temperate grasses that share similar habitats and environmental gradients. The large reduction in belowground production, crown biomass and crown reserves in response to climate treatments is likely to have contributed to the reduced resistance and resilience of aboveground production to climate extremes. Despite treatment-related shifts in root traits to support greater resource acquisition, the potential benefits of increased SRL were not enough to counteract the negative impacts of combined warming and drought effects on aboveground production. Furthermore, the trade-offs between belowground production and root trait plasticity highlighted the importance of biomass allocation belowground. This study provides key insights into the belowground responses of a single species to climate extremes, which may differ from (and are challenging to assess in) mixed plant communities.

## Supplementary data

The following supplementary data are available at *JXB* online.

Fig. S1. Climate treatments and environmental conditions under each climate treatment during the experimental period.

Table S1. Summary statistics for post-drought recovery biomass from Wilcoxon signed rank test.

erad021_suppl_supplementary_fig_S1_table_S1

## Data Availability

Data from this study are available at https://doi.org/10.6084/m9.figshare.19387637.v1 (Chandregowda *et al.*, 2022c).

## Abbreviations

ANPP: aboveground net primary production (g m^–2^)
BNPP: belowground net primary production (g m^–2^)
*f*_BNPP_: the fraction of primary production allocated belowground
N: nitrogen (%)
NSC: non-structural carbohydrate
RMF: root mass fraction
RTD: root tissue density (g root dry mass cm^–3^ root volume)
SRL: specific root length (m root length g^–1^ root dry mass).
