# Supplementary material for: Belowground carbon allocation, root trait plasticity, and productivity during drought and warming in a pasture grass
Source: J Exp Bot. 2023 Jan 14;74(6):2127–45. doi: 10.1093/jxb/erad021 (PMC10084810; doi:10.1093/jxb/erad021)
Supplement: erad021_suppl_supplementary_fig_S1_table_S1 [file erad021_suppl_supplementary_fig_s1_table_s1.pdf]

Supplementary data (Chandregowda et al.)

Belowground carbon allocation, root trait plasticity and productivity during drought and warming in a pasture grass

Manjunatha H. Chandregowda[*1], Mark G. Tjoelker[1], Elise Pendall[1], Haiyang Zhang[1], Amber C. Churchill[1, 2], Sally A. Power[1]

[1]Hawkesbury Institute for the Environment, Western Sydney University, Locked Bag 1797, Penrith, NSW, 2751, Australia

[2]Department of Ecology, Evolutionary Biology and Behaviour, University of Minnesota, 140 Gortner Laboratory, 1479 Gortner Ave., St. Paul, MN 55108 USA

[*]Corresponding author

Email address: M.Chandregowda@westernsydney.edu.au

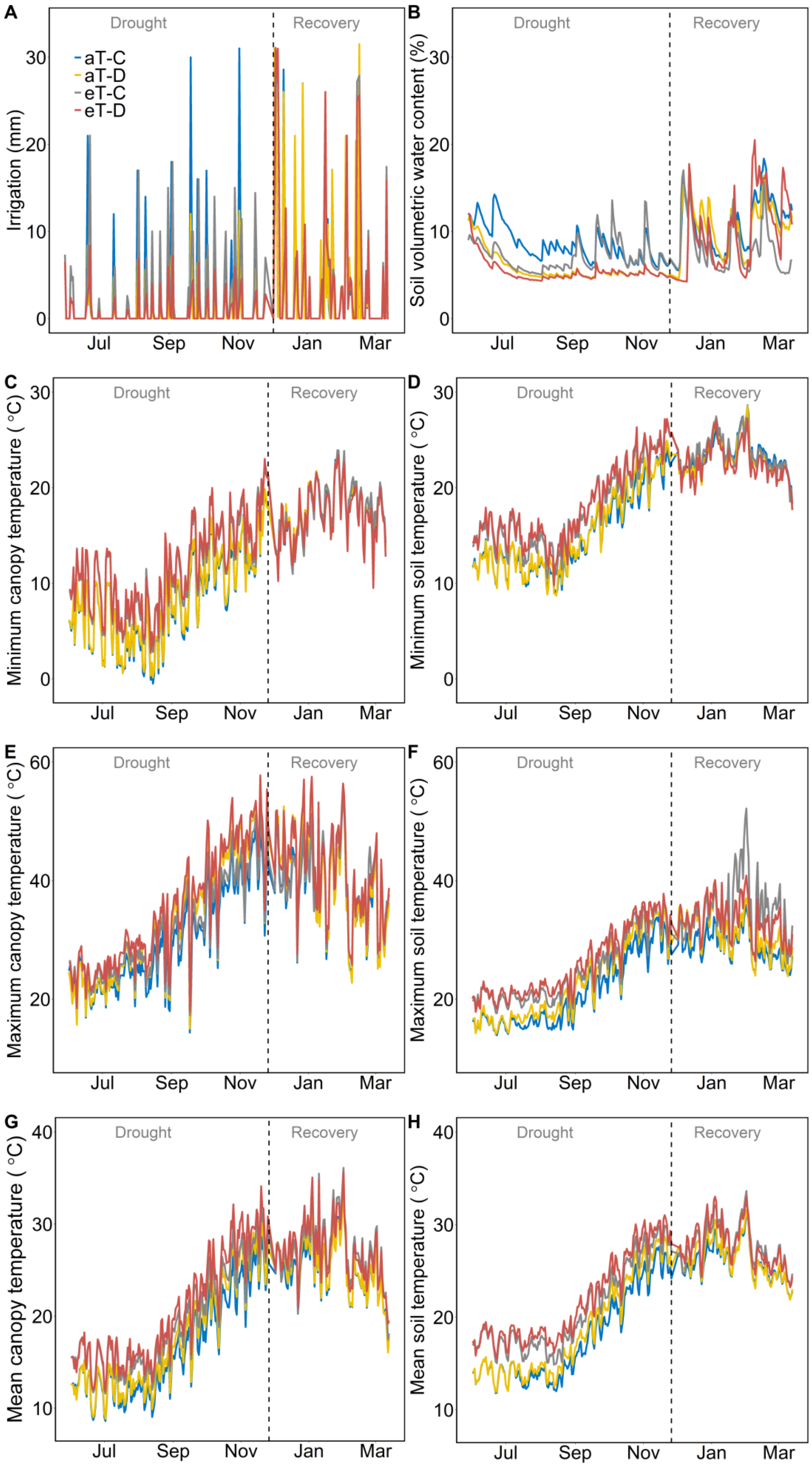

A
Drought
Recovery
aT-C
aT-D
eT-C
eT-D
Irrigation (mm)
0
10
20
30
Jul
Sep
Nov
Jan
Mar
B
Soil volumetric water content (%)
C
Minimum canopy temperature (°C)
D
Minimum soil temperature (°C)
E
Maximum canopy temperature (°C)
F
Maximum soil temperature (°C)
G
Mean canopy temperature (°C)
H
Mean soil temperature (°C)

Fig. S1 A Irrigation water inputs and the corresponding **B** soil volumetric water content (0-15 cm depth) measured during the cool-season drought period (between 1 June 2019 to 30 November 2019) and the following summer recovery period (between 1 December 2019 to mid-March 2020) in both ambient and elevated temperature treatments. Canopy temperature **C** minimum, **E** maximum and **G** mean, soil temperature **D** minimum, **F** maximum and **H** mean recorded in aT-C (ambient temperature-control), aT-D (ambient temperature-drought), eT-C (elevated temperature-control), and eT-D (elevated temperature-drought) plots both during cool-season drought and summary recovery period. Mean values across plots (n = 6) are shown.

Table S1. Summary statistics for post-drought recovery biomass from Wilcoxon signed rank test. Temperature and water represent climate treatments, and W indicates the Wilcoxon signed rank test statistic.

**Response variable**	**Temperature**	**Water**	**W**	***p*-value**
	Ambient temperature	Control-Drought	16	0.54
Aboveground biomass recovery	Elevated temperature	Control-Drought	17	0.39
